# Startup performance of microbial electrolysis cell assisted anaerobic digester (MEC-AD) with pre-acclimated activated carbon

**DOI:** 10.1016/j.biteb.2018.12.007

**Published:** 2019-02

**Authors:** Suyun Xu, Yuchen Zhang, Liwen Luo, Hongbo Liu

**Affiliations:** School of Environment and Architecture, University of Shanghai for Science and Technology, Shanghai 200093, China

**Keywords:** Activated carbon, Direct interspecies electron transfer, *Geobacter*, Hydrogenotrophic methanogens, Volatile fatty acids

## Abstract

The feasibility of using pre-acclimated activated carbon to start up microbial electrolysis cell assisted anaerobic digester (MEC-AD) has been testified in this study. Two identical lab-scale digesters were separately packed with granular activated carbon (GAC) and powered activated carbon (PAC), which were initially acclimated as anaerobic digester and then transferred to MEC-AD. When a voltage of 0.5 V was applied, increased methane generation and substrate removal rates were observed. Hydrogenotrophic methanogens predominated in both digesters before and after transition, indicating that the pre-cultured microbial community on carbon materials could provide necessary microbiome favorable for starting up MECs. Although a low abundance of *Geobacter* was detected in inoculum, a rapid propagation could be realized when reactors were subjected to the electro-stimulation. The abundance of *Methanosarcina* closely attached to PAC was four times than that of GAC, which might be partially contributed to the improved resilience of anaerobic digester subjected to electro-stimulation.

## Highlights

• Pre-acclimated PAC/GAC are favorable for starting up MEC-AD.
• Methane yield was increased by ~30% when transferring AD to MEC-AD.
• Abundance of electroactive bacteria on pre-enriched PAC was higher than GAC.
• The rapid propagation of Geobacter was found in MEC-AD.

## 1 Introduction

Anaerobic methanogenesis is the way of sustainable treatment of high concentration organic wastes. In anaerobic digestion (AD) process, the degradation of various natural polymers such as polysaccharides, proteins and lipids to CO_2_ and CH_4_ involves a complex microbial community (Xu et al., 2017). Syntrophic associations are essential metabolic ways for different species, such as the fatty acid degraders and the methanogens. But, the balance between microbial communities are easily interfered such as due to the local accumulated acids and partial hydrogen pressure (*P*_*H2*_) etc. (Dahiya et al., 2015). To overcome these limitations, several methods have been attempted in recent years (Aryal et al., 2018). Direct interspecies electron transfer (DIET) has been built up in anaerobic digester fed with magnetic particles, in which way, the electron transfer rate is considered to be 10^6^ faster than interspecies hydrogen transfer (IHT) (Cruz Viggi et al., 2014). DIET pathway can also be established in digesters with carbon materials such as granular activated carbon (GAC), biochar and carbon fiber etc., which lead to better persistence to higher organic loading rate (OLR) compared with conventional digester (Dang et al., 2016; Zhao et al., 2016).

Another new type of methanogen generating reactor, namely microbial electrolysis cell (MEC) is attracting more and more attention in recent years because of its flexible performance on enhancing degradation of organics and methane/hydrogen production (Pasupuleti et al., 2015). MECs use microbes grown on one or both electrodes to produce gaseous fuels with the addition of external electrical input. It is originally used to produce hydrogen, but in the practice it is found that the cathodic reduction of CO_2_ to CH_4_ is inevitable and then more researchers began to explore the possibility of enriched methane generation from MECs (Villano et al., 2010; Aryal et al., 2018). The energy efficiency can even reach to 80% via hydrogenotrophic methanogenesis (Cheng et al., 2009). Our previous study revealed that the degradation rates of acetate and butyrate were significantly increased in MECs with bioanode of graphite felt under the applied voltage of 0.5 V, as compared to reactors without power supply (Luo et al., 2018).

For MECs, *Geobacter* populations are one of the dominated microorganisms (Luo et al., 2018; Yu et al., 2018). The growth of exoelectrogenic bacteria, especially *Geobacter* species, is boosted in MEC-AD, which results in the accelerated decomposition of substrates (Speers et al., 2014). *Geobacter* species can anaerobically utilize a wide range of organic compounds as substrates, such as VFAs and aromatic hydrocarbons; meanwhile it also can cooperate with other fermentative bacteria or syntrophic VFAs degrading bacteria such as *Smithella*, *Bifidobacterium* and *Clostridium* etc., coexisting in MEC-AD (Li et al., 2016; Lovley et al., 2011). Nevertheless, the effective methods to enrich electrotrophic bioelectrode communities and quick startup of MECs have not been well studied yet. The effluent from microbial fuel cell (MFC), bog and other resources containing exoelectrogenic bacteria are usually required as inoculum for MECs (Fu et al., 2015). In fact, during the trials to promote DIET in anaerobic digesters, *Geobacter* and *Methanosaeta* species were found to be enriched on the surface of biochar and other conductive materials, and this change contributed to the enhancement of methane production (Yan et al., 2017; Zhao et al., 2017). LaBarge et al. had verified the pre-acclimation of electroactive communities on GAC with different organic substrates (LaBarge et al., 2017). Thus, it is possible to use the pre-enriched culture on activated carbon as inoculum for starting up MEC-AD.

Hence, the objective of this study is to evaluate the feasibility of startup of methane generating MEC-AD by using activated carbon packed anaerobic digesters that have been pre-acclimated to the synthetic brewery wastewater. The transition of microbial community enriched on granular activated carbon (GAC) and powered activated carbon (PAC) has been characterized. Furthermore, the performance of MEC-AD subjected to varied applied voltage has been examined by evaluating the substrate degradation rates and methane yield.

## 2 Material and method

### 2.1 Synthetic wastewater and inoculation

The volatile suspended solids (VSS) concentration of sludge was adjusted to 6.0 g/L in reactors, and the ratio of VSS to total suspended solids (TSS) is 66%. Synthetic brewery wastewater was fed to anaerobic digesters as the substrate (Xu et al., 2015). The concentrations of ethanol and glucose were 28.2 g/L and 18.0 g/L, respectively. Other supplements included 2.59 g urea, 0.20 g yeast extract, 1.91 g K_2_HPO_4_, 1.24 g KH_2_PO_4_ and 2 mL trace element solution per liter. The total COD and total organic carbon (TOC) concentration of original solution for synthetic wastewater were 65.3 g/L and 22.5 g/L, respectively.

### 2.2 Experimental equipment and operation

Two identical lab-scale upflow anaerobic digesters (internal diameter of 160 mm and height of 360 mm) were used in this study. Their working volume is 5.6 L. Two different particle sizes of coal-based AC, i.e. 10–20 mesh (0.84–2.00 mm) of granular activated carbon (GAC) and 80–100 mesh (75–177 μm) of powered activated carbon (PAC) were added into R1 and R2, respectively, each of which received 5 g/L. Reactors were placed in a temperature-control incubator at 35 ± 2 °C without light. Reactors were initially cultured and operated as conventional AD digesters, whose starting up performance had been reported previously (Xu et al., 2015).

**Fig. 1:**
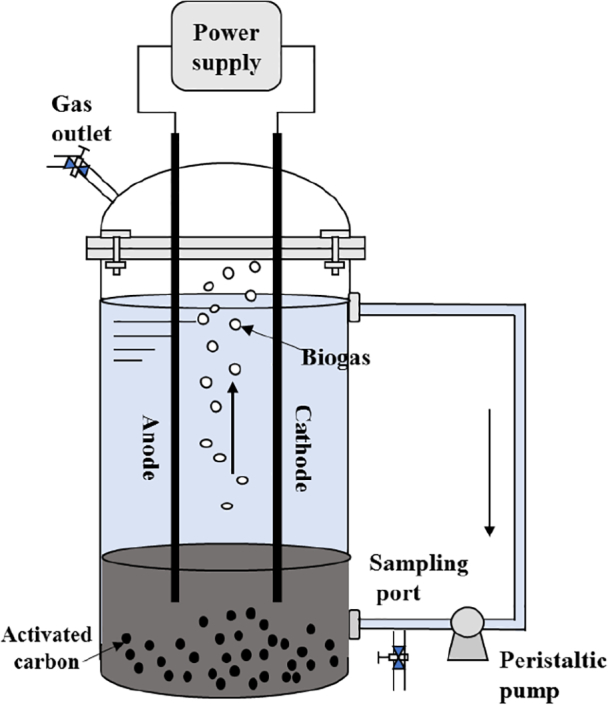
Schematic diagram of the MEC-AD combined system.

After that, the OLR and hydraulic retention time (HRT) were maintained at 5.8 g COD L/d and 5.6 d, respectively. The MECs assisted anaerobic digester (MEC-AD) reactors were constructed by placing a pair of graphite rod electrodes (ø6 mm × L300 mm) in R1 and R2 respectively, as shown in Fig. 1. Titanium wires are used to connect the electrodes with direct current power supply. The applied voltage is adjusted to be 0.5 V at Stage I and 1.0 V at Stage II, respectively. At Stage III, the intermittent electro-stimulation at 1.0 V is applied, i.e. turn-on and turn-off at the interval of 24 h. The details of stage design are shown as Table 1.

**Table 1:** The operational stages of R1 and R2.

Stage	Time (d)	OLR (g COD L/d)	Voltage (V)	
			R1	R2
I	1–80	5.8	0.5	0.5
II	81–95	5.8	1.0	1.0
III	96–110	5.8	1.0/0	1.0/0

### 2.3 Analytical methods

Each reactor was sampled to monitor the variations of pH, total organic carbon (TOC) and volatile fatty acids (VFAs). VFAs were analyzed by using a high performance liquid chromatography (Waters 2695/2489, Waters, America). TOC was detected by total organic carbon/total nitrogen analyzer (Multi N/C 3100, Jena Co., Germany). Biogas compositions (CH_4_, CO_2_ and H_2_) were determined by a gas chromatograph (GC9890B, Shanghai Linghua Co., China).

The sludge samples were collected from R1 and R2 before electric stimulation (i.e. US_R1 and US_R2), and after the acclimation to the applied voltage of 0.5 V (i.e. eUS_R1 and eUS_R2). To study the spatial distribution of bacteria and archaea on AC, the microorganisms in the sludge samples were separated into three fractions, i.e. suspended (S), loosely attached (L) and tightly adsorbed (T) fractions (Luo et al., 2015). As the surface area of graphite rod electrode is quite small (~0.011 m^2^) and the attached biomass is expected, no sampling was performed on it. Then the total DNA of three fractions were extracted and analyzed by using a high-throughput pyrosequencing platform (Illumina Miseq PE300, Majorbio Ltd. Co). The primers 515F (5′-GTG CCA GCM GCC GCG GTAA-3′) and 806R (5′-GGA CTA CHV GGG TWT CTA AT-3′) were used to simultaneously obtain bacterial and archaeal information (Bates et al., 2011).

## 3 Results and discussions

### 3.1 The pH and TOC variation

**Fig. 2:**
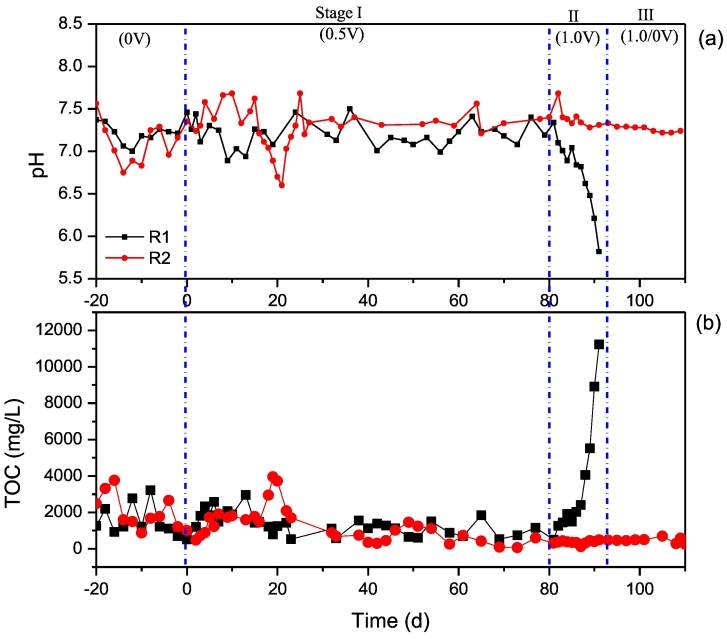
Variations of (a) pH and (b) TOC in effluent from R1 and R2.

Fig. 2 presents the changes of TOC and pH in effluent from R1 and R2. In the initial 10 days of Stage I, relatively acidic environment and higher TOC concentrations were observed in effluents of R1 when compared to R2. In the following 10 days, a short period of instability was performed in R2, in which pH decreased to 6.5 and TOC concentration increased to 4000 mg/L. Nevertheless, the pH in R2 was recovered soon to the optimum range after that. Overall, the values of pH and TOC concentration were instable at initial period with electro-stimulation; after 20 days, stable values and similar tends of pH and TOC concentrations were observed in R1 and R2. When the applied voltage increased to 1.0 V at Stage II, an irreversible deterioration was observed in R1 due to the constant acid accumulation which was presented as acidic pH and cumulative TOC concentration.

In general, pH is recognized as one important indicator for methanogenic performance, the optimum value for the growth of methanogenic bacteria in anaerobic digestion is around 6.6–7.6 (Reungsang et al., 2012). According to the results of R1 and R2 during Stage I and II, a stable methanogenic process would be disrupted, especially on the initial period, when adopted the external electro-stimulation. Although electro-stimulation would affect the degradation performance, R2 had performed a self-recovery ability to resist the environmental change. In addition, it was showed that values of pH and TOC concentration in R2 were stable during the whole process from Stage I to III. Therefore, the substrate degradation in UASB would be influenced by additional electricity, but PAC had better capability to maintain methanogenic process than GAC facing electro-stimulation.

### 3.2 The VFAs variations

**Fig. 3:**
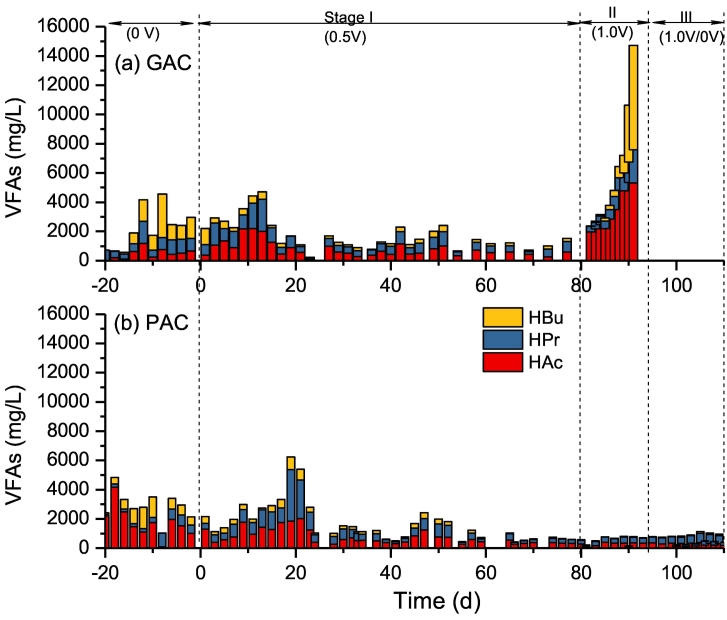
The VFAs variation of R1 (GAC) and R2 (PAC) during different stages.

The VFAs concentration was shown in Fig. 3. At stage I, the variations of VFAs in R1 and R2 were quite similar, which average concentration of effluent was close to 3000 mg/L at the beginning of adopting electro-stimulation. After 20 days, the accumulated concentration of VFAs was gradually reduced and maintained at a relative stable level, nevertheless it was lower in R2 (~500 mg/L) as compared to R1 (~1100 mg/L). In addition, it clearly showed that R1 was undergoing acidification when increased applied voltage to 1.0 V, as supported by the higher cumulative VFAs concentrations which even increased up to 14 g/L. This result was in accordance to the acidic pH (Fig. 2a). It indicated that PAC had better capability to convert substrate and maintain digestion performance in this system comparing to GAC. Compared to GAC, PAC exhibits higher surface area for the absorption of soluble low molecular weight organic compounds as compared to GAC (Akram and Stuckey, 2008; Xu et al., 2018). On a certain aspect, the lower total VFAs concentration in R2 might be related to the absorption effect of PAC. Meanwhile, PAC is more favorable for the colonization of specific bacteria. Previous study found that higher abundance of methanogens and syntrophic VFAs-oxidizing bacteria were attached on PAC, contributing to the enhanced degradation of VFAs (Xu et al., 2015).

Acetate, propionate, and butyrate were the predominant VFAs components of acidogenesis, which proportion would influence the methane production. During acidogenesis, acetate could be produced by the oxidation process of propionate and butyrate. As shown in Fig. 3, different kinds of acid were accumulated at instable period with electro-stimulation. Light propionate accumulation was observed in R1 and R2 at Stage I, while butyrate and acetate were significantly accumulated in R1 at Stage II. At the end of Stage II, the concentrations of HAc, HBu and HPr in R1 were up to 5322, 2258 and 7147 mg/L, respectively; whereas, HAc and HPr predominated in R2 and the total concentration of VFAs in effluent was <500 mg/L. As discussed above, the instable performance at Stage I could be recovery, but the deterioration in R1 at Stage II was irreversible because of the accumulation of acetate which could be directly utilized by methanogens. These results indicate that the R2 reactor added PAC has better coping capacity than R1 when sudden change the applied voltage.

### 3.3 Methane yield changes

**Fig. 4:**
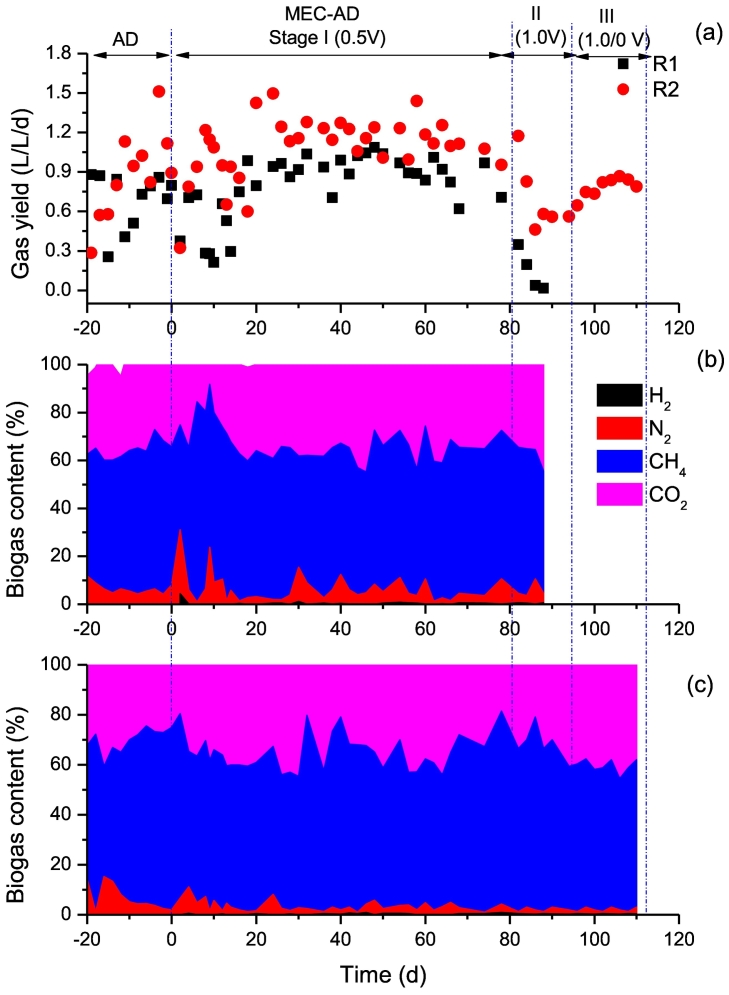
Variations of biogas generation during different stages: (a) volumetric gas yield, (b) biogas content from R1 and (c) biogas content from R2.

Methane yield in anaerobic digestion is a good indictor showing electron transfer efficiency from substrate to methane (Zhao et al., 2014). The methane generation of different stages was recorded in terms of volumetric biogas yield (L/L/d) and biogas content (%), as shown in Fig. 4. Initially, a relatively low voltage of 0.5 V was applied to R1 and R2, which was maintained for a long period i.e. 80 days to ensure the acclimation of symbiotic microorganism. Before connecting to the power supplier, the average volumetric methane yields from R1 and R2 were 0.68 and 0.88 L/L/d, which decreased slightly in the beginning of Stage I and then increased to a steady state, i.e. 0.91 and 1.16 L/L/d. In average, about 30% of increment was found for the methane yield when transferring AD to MEC-AD. Nevertheless, the changes of CH_4_ and CO_2_ content in biogas were insignificant when the operation mode changed from AD to MEC-AD with small electro-stimulation. The average CH_4_ concentration of R1 and R2 were 58.0% and 62.3% in the AD mode, which slightly changed to 58.4% and 63.1% at the Stage I of MEC-AD mode.

At stage II, when the applied voltage was elevated to 1.0 V, the methane generation of R1 was sharply decreased and almost ceased at Day 94. It is corresponding to the accumulation of VFAs (>10 g/L) and the drop of pH (<6.0), as shown in Fig. 3, Fig. 2. For R2, the decrease of methane yield was also found at Stage II, nevertheless it tends to be restored when changing the voltage supply method at Stage III. Finally, its biogas yield decreased by 20% and CH_4_% slightly decreased to 57.3% as compared to Stage I.

Obviously, methane generation of R1 and R2 were both improved by applying a low voltage of 0.5 V. However, the performance of R2 with PAC was better than R1 with GAC. It also found that the maximum current in R2 (6.1–7.9 mA) was higher than R1 (2.7–4.1 mA), which is correlated to the trend of methane yield. Previous studies have shown that methanogens generate methane using electric current from cathodes (Cheng et al., 2009) and GAC can stimulate current generation by exoelectrogenic bacteria (Liu et al., 2014). In this experiment, the higher electric current of reactor with PAC might be correlated to the selectively enriched electroactive bacteria, which help participated in the degradation of VFAs or facilitating DIET for methane generation (Zhao et al., 2014).

### 3.4 Comparison on the microbial community structure

#### 3.4.1 Archaeal community structure

**Fig. 5:**
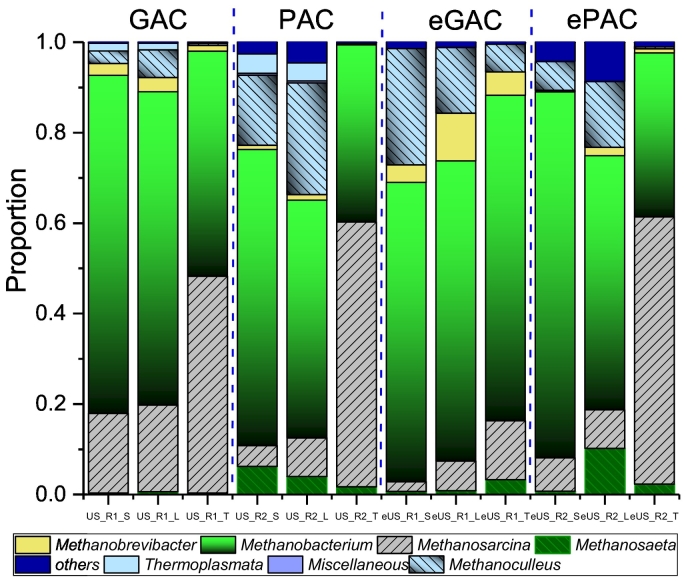
Archaeal taxonomic distributions.

As shown in Fig. 5, the main methanogenic species in R1 and R2 are *Methanosaeta*, *Methanosarcina*, *Methanobacterium*, *Methanobrevibacter* and *Methanoculleus*. Overall, *Methanobacterium*, one of the hydrogenotrophic methanogens, predominated in the digester, which accounts for 36.2%–80.9% of the total archaea. Meanwhile, *Methanosaeta*, the typical acetoclastic methanogens, which only takes up a low content of 0.3–10.2% in the system.

Comparatively, *Methanosarcina* tends to closely attach to the surface of activated carbon, i.e. the tight phase (T). When subjected to electric stimulation, the abundance of *Methanosarcina* in R1_T decreased from the original value of 47.9% to 13.0%, whereas its abundance maintained at a similar level in R2_T, i.e. 58.6% (before) and 59.1% (after). Correspondingly, the abundance of *Methanobacterium* in R1_T increased from 49.8% to 71.9%. *Methanoculleus* species are known to dominate reactors with high concentrations of VFAs (Franke-Whittle et al., 2014), which also can utilize hydrogen and carbon dioxide/formate as substrate to produce methane (Zhao et al., 2017). Its abundance significantly increased from 2.7% and 6.1% to 25.7% and 14.5% in the suspended phase (S) and loosely attached phase (L) of GAC in R1 after electric stimulation. It indicates that the advantage of hydrogenotrophic methanogenesis in R1 is more obvious when subjected to electric stimulation. Whereas the variations of archaeal community in R2 are less than R1. *Methanosarcina* and *Methanobacterium* co-existed in the “T” fraction, meanwhile *Methanobacterium* still predominated in the “S” and “L” fractions after the transition to MEC-AD.

#### 3.4.2 Bacterial communities structure

**Fig. 6:**
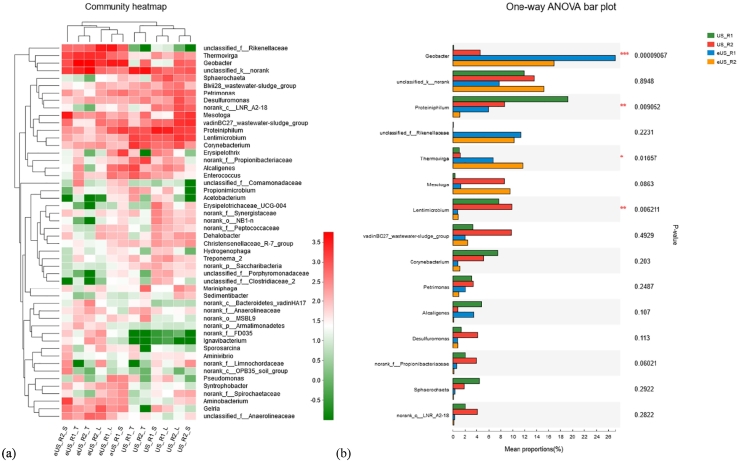
(a) Bacterial community heatmap at genus level, (b) one-way ANOVA analysis.

As shown in Fig. 6a, the relative bacterial community abundance is given on the genus level. Comparing with the results of our previous study (Xu et al., 2015), the strategy of electro-stimulation brought big changes on bacterial structure, such as the significant increase on the abundance of *Geobacter* species, and the decrease of *Proteiniphilum*. *Proteiniphilum* is reported to be capable of converting pyruvate to acetic acid and CO_2_ (Chen and Dong, 2005), which abundance decreased significantly in both reactors (*P* = 0.009) when subjected to external power supply, as shown in Fig. 6b. Whereas the abundance of *Geobacter* increased from original abundance of <10% to >40% (of total bacteria). The predominant species on AC is *Geobacter* sp., which are of great interest for environmental and biotechnology applications as they can be capable of oxidizing acetate and then participant in DIET with *Methanosaeta* sp. via PAC (Liu et al., 2012). Comparatively, before electric stimulation the *Geobacter* pre-enriched in R2 packed with PAC is quite higher than R1 with GAC. Moreover, aparting from *Geobacter*, *Pseudomonas* species are also recognized as electrogenic bacteria responsible for converting VFAs to electric current (Logan, 2009). They might also participate in direct DIET.

Fig. 6 also tells that the abundance of *Aminobacterium* and *Mesotoga* sp. in R2 are much higher than R1. *Aminobacterium* is a kind of amino-acid fermenting bacterium. It is reported that *Aminobacterium* and the hydrogenotrophic methanogens could form a symbiotic relationship in coculture, e.g. *Methanobacterium* (Baena et al., 1998). Its abundance increased after electric stimulation, and the higher abundance is found in the “S” fraction of eUS_R2. *Mesotoga* is a mesophilic member of *Thermotogales*, which is capable of oxidizing sugars including cellobiose and xylose to produce acetate, sulfide and carbon dioxide (Nesbo et al., 2010), and hydrogen gas (Conners et al., 2006). The high abundance of *Mesotoga infera* was detected in the anaerobic digester of cellulosic material, indicating this participant could provide positive effects on the methane yield (Lee et al., 2017). Thus, the key reason for running stable condition in R2 could be attributed to the closely built up syntrophic relationship among VFAs degrading bacteria, *Geobacter* and methanogens, i.e. *Methanosarcina or Methanosaeta*.

## 4 Discussion

In general, recent researches on methane-producing MECs mainly focused on the aspects of the control of reactor type (Sleutels et al., 2011), electrode type (Bajracharya et al., 2015; Sasaki et al., 2011) and reaction conditions (Ding et al., 2018; Ding et al., 2016). Nevertheless, the strategy for the fast startup of MECs has limited attention. This study has investigated the transition of microbial community subjected to electric stimulation, as well as the reactors' performance, i.e. the substrate degradation rates and methane yield.

In most MECs with methane generation, hydrogenotrophic microorganisms are always more abundant than acetoclastic methanogens, although acetate is fed as the substrate, among which *Methanobacterium* is frequently reported (Siegert et al., 2014). Inocula obtained from a natural bog sediment with high quantities of hydrogenotrophic methanogens showed higher methane generation in MECs than reactors inoculated with anaerobic digester sludge with mostly acetoclastic methanogens (LaBarge et al., 2017). In this study, *Methanobacterium* and *Methanosarcina* predominated in the inoculum. When subjected to electro-stimulation, the proportion of hydrogenotrophic microorganisms in MEC-AD digesters tends to increased slightly, indicating that the pre-enriched microbial community on the activated carbon may contribute to the smooth transition. Whereas, the effect of electro-stimulation on bacterial community variations is more distinct, as evidenced by the results of redundancy analysis (RDA) in Supporting information. Comparatively, the higher abundance of *Methanosarcina* selectively adhered to PAC as compare to GAC, and it was quite stable after electro-stimulation. *Methanosarcina* can produce CH_4_ through three metabolic pathways using H_2_/CO_2_, acetate and methylated one‑carbon compounds (De Vrieze et al., 2012), which can also potentially participant in DIET with *Geobacter* (Rotaru et al., 2014) and other bacteria (Ying et al., 2017). The improved resilience of R2 could be highly correlated to the higher abundance of *Methanosarcina* on pre-cultured PAC.

The optimization of external energy (current or voltage) plays a key role in product formation (CH_4_ generation) and COD degradation (Zhao et al., 2016). Previous researches on optimal voltage were limited to reactor types, and different reactor configurations resulted in different optimal voltages. Although the optimal voltage might be different, a similar conclusion can be obtained that the COD removal efficiency, the maximum current and methane generation firstly increased and then decreased with the voltage ranging from 0 to 2 V (Choi et al., 2017; Ding et al., 2018; Ding et al., 2016). Ding et al. found that COD removal efficiency and methane yield were increased when the applied voltage is lower than 0.8 V; the higher plasmatorrhexis and lower growth and metabolism were observed at voltages higher than 0.8 V (Ding et al., 2016). Another study reported that 0.6 V was proved to be the best value as applied voltage for COD removal (Ding et al., 2018). Choi et al. reported the maximum current and methane generation in MEC were obtained at an applied voltage of 1.0 V (Choi et al., 2017). In present study, the higher methane yield was obtained at 0.5 V in MEC-AD with exogenous material PAC without changing the configuration of the reactor. When increasing the voltage to 1.0 V, instability occurred in the system. This might be attributed to the harmful effects of electricity on living organism, such as plasmatorrhexis and limited bacterial growth and metabolism. Nevertheless, the performance of R2 tended to be recovered soon, whereas R1 presented further deterioration. The conductivity characteristic of PAC and the increased biomass might be the key factor (Liu et al., 2014). It has been proved that the defined *Geobacter* and *Methanosaeta* or *Methanosarcina* species could exchange electrons via DIET (Rotaru et al., 2014), which related to energy conservation mechanisms (Nagarajan et al., 2013). The higher abundance of *Methanosarcina* and *Geobacter* were found to be clustered on the surface of PAC, which might help to improve the resilience of digester subjected to the higher voltage. Unfortunately, it is difficult to explain the reasons clearly based on the current results, which will be further explored in future study.

Up till now, the effect of changing power supply mode on the biotic efficiency of MEC is less investigated. Due to the economy of renewable energy, the excess electricity can be injected to the MEC system to harvest storable energy, however it might be suffered from fluctuations and even interruptions. Finkelstein et al. (2006) revealed that the microorganisms of bioanode could adapt their electron transferring system to a level even opening the circuit. Considering that some substrates still can be used by the biocatalyst on the electrodes under the open circuit, more studies are needed to evaluate the effect of power supply mode on the reactor's performance. In present study, the interruption period of 24 h was adopted in R2 at Stage III, and the response of substrate consumption rate and methane yield were compared between the on and off periods. As presented in Figs. 3b and 4c, their variations were insignificant, indicating that the microbial community could maintain itself without requiring external energy supply during the off periods. Anzola-Rojas et al., 2018a, Anzola-Rojas et al., 2018b also revealed that microbial community on MES was resilient and able to recover the electro-autotrophic activity despite the duration of current supply interruptions. Nevertheless, reversed pathway might occur when a longer period of power supply interruption was applied, i.e. 64 h (Anzola-Rojas et al., 2018b). Such resilience of electrotrophic microorganisms could overcome the electrical power fluctuations and reinforce the opportunity to couple bioelectrochemical systems to renewable energy. At last, it's probably worth pointing out that the contribution of electrotrophic methanogenesis to the overall methane generation in present MEC-AD system is still not clear, which requires further detailed study to illuminate the relationship.

## 5 Conclusions

In present study, results indicate that the predominated hydrogenotrophic methanogens and in the anaerobic reactors pre-cultured with PAC and GAC, which could provide necessary microbiome to startup MECs. Although the abundance of *Geobacter* in inoculum is not high, a rapid propagation could be realized when the digesters subjected to a low voltage of 0.5 V. Comparatively, PAC is superior to GAC on enhancing biomethane generation process and keeping the stability of MECs. The higher abundance of *Methanosarcina* in the “T” fraction of PAC reactor might be highly correlated to the improved resilience of anaerobic digester subjected to electro-stimulation.

The following is the supplementary data related to this article.

Fig. S1 Redundancy analysis (RDA) analysis on bacterial community. Environmental factors include: applied voltage (eV), effluent pH (pH) and effluent VFAs concentration (VFAs). Fig. S1

## Conflict of interest

We declare that we have no financial and personal relationships with other people or organizations that can inappropriately influence our work, there is no professional or other personal interest of any nature or kind in any product, service and/or company that could be construed as influencing the position presented in, or the review of, the manuscript entitled “Startup performance of microbial electrolysis cell assisted anaerobic digester (MEC-AD) with pre-acclimated activated carbon”.
