# Supplementary material for: Startup performance of microbial electrolysis cell assisted anaerobic digester (MEC-AD) with pre-acclimated activated carbon
Source: Bioresour Technol Rep. 2019 Feb;5:91–8. doi: 10.1016/j.biteb.2018.12.007 (PMC6524652; doi:10.1016/j.biteb.2018.12.007)
Supplement: Fig. S1 — Redundancy analysis (RDA) analysis on bacterial community. Environmental factors include: applied voltage (eV), effluent pH (pH) and effluent VFAs concentration (VFAs). [file mmc1.docx]

**Supporting information**

**For**

# Startup performance of microbial electrolysis cell assisted anaerobic digester (MEC-AD) with pre-acclimated activated carbon

# Suyun Xu*, Yuchen Zhang, Liwen Luo, Hongbo Liu**

School of Environment and Architecture, University of Shanghai for Science and Technology, Shanghai 200093, China

* Corresponding author. E-mail: xusy@usst.edu.cn, Tel (Fax): +8621 55275979

**Common corresponding author: Email: [Liuhb@usst.edu.cn](mailto:Liuhb@usst.edu.cn)


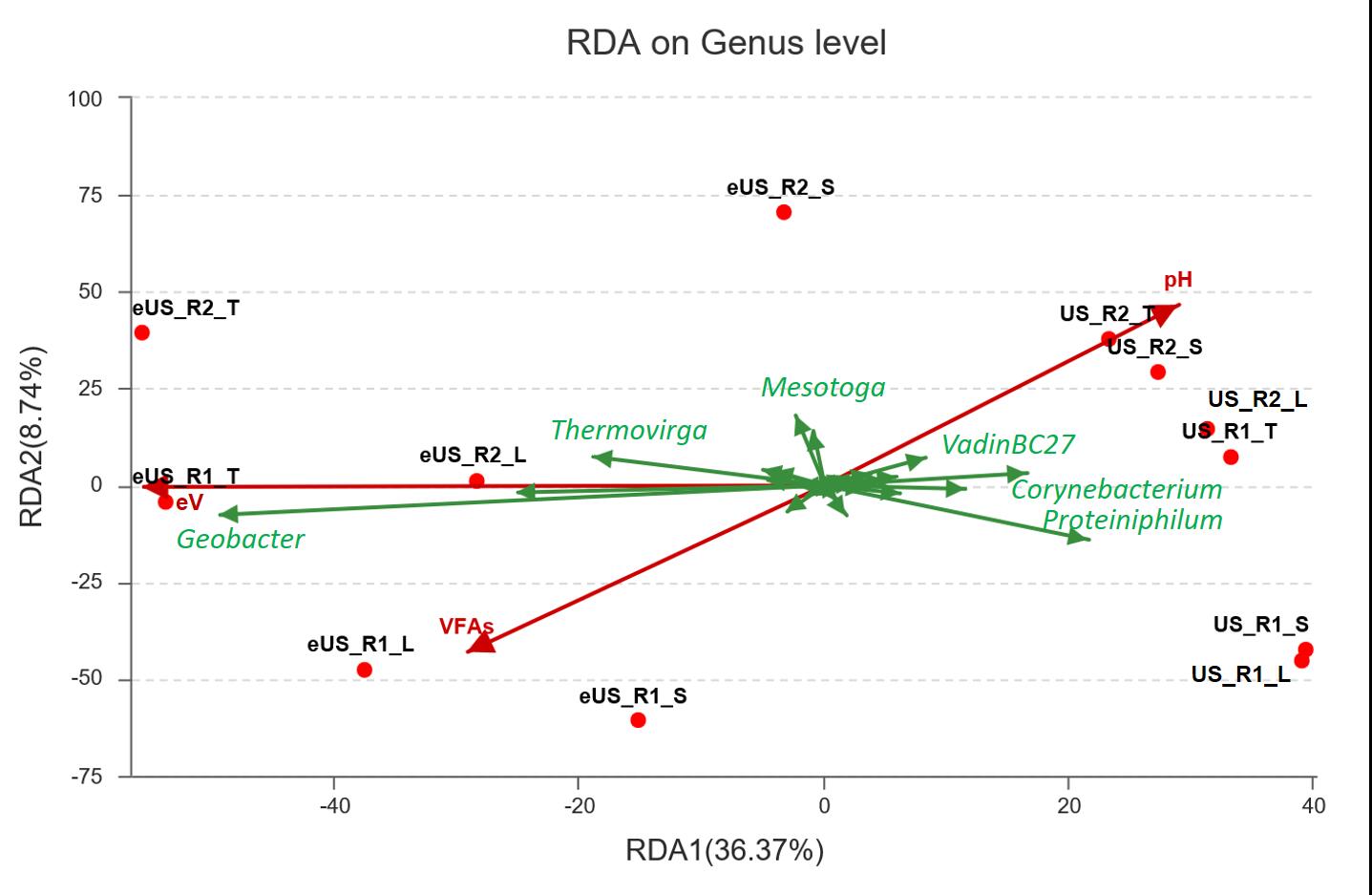


Fig. S1 Redundancy analysis (RDA) analysis on bacterial community.

Environmental factors include: applied voltage (eV), effluent pH (pH) and effluent VFAs concentration (VFAs).
